# Developmental vitamin D-deficiency produces autism-relevant behaviours and gut-health associated alterations in a rat model

**DOI:** 10.1038/s41398-023-02513-3

**Published:** 2023-06-14

**Authors:** Man Kumar Tamang, Asad Ali, Renata Nedel Pertile, Xiaoying Cui, Suzy Alexander, Marloes Dekker Nitert, Chiara Palmieri, Darryl Eyles

**Affiliations:** 1 Queensland Brain Institute, The University of Queensland, Brisbane, Australia; 2 Queensland Centre for Mental Health Research, Wacol, Australia; 3 School of Chemistry and Molecular Bioscience, The University of Queensland, Brisbane, Australia; 4 School of Veterinary Science, The University of Queensland, Gatton, Australia

**Keywords:** Molecular neuroscience, Neuroscience

## Abstract

Developmental vitamin D (DVD)-deficiency is an epidemiologically established risk factor for autism. Emerging studies also highlight the involvement of gut microbiome/gut physiology in autism. The current study aims to examine the effect of DVD-deficiency on a broad range of autism-relevant behavioural phenotypes and gut health. Vitamin D deficient rat dams exhibited altered maternal care, DVD-deficient pups showed increased ultrasonic vocalizations and as adolescents, social behaviour impairments and increased repetitive self-grooming behaviour. There were significant impacts of DVD-deficiency on gut health demonstrated by alterations to the microbiome, decreased villi length and increased ileal propionate levels. Overall, our animal model of this epidemiologically validated risk exposure for autism shows an expanded range of autism-related behavioural phenotypes and now alterations in gut microbiome that correlate with social behavioural deficits raising the possibility that DVD-deficiency induced ASD-like behaviours are due to alterations in gut health.

## Introduction

Autism Spectrum Disorders (ASD) are neurodevelopmental disorders demonstrating early childhood onset of social, communication and behavioural impairments [1]. Epidemiological studies have suggested an association linking vitamin D-deficiency during pregnancy and infancy (referred to as Developmental vitamin D (DVD)-deficiency) with risk of autism in the offspring [2–5]. However in populations with a high proportion of vitamin D sufficiency this relationship has become difficult to test [6, 7] indicating ASD-risk for DVD-deficiency operates via a clinical threshold rather than as a continuous measure [8].

Our laboratory was the first to establish a rat model of DVD-deficiency to examine brain-related outcomes in offspring [9]. Since then, we (and others) have published numerous studies showing the impact of DVD-deficiency on brain development [10]. Recent behavioural and molecular studies in DVD-deficient rats from our laboratory have revealed ASD-related behavioural alterations [11], placental immune dysregulation [12] and elevated testosterone levels in male embryonic brains [13], all potentially relevant to autism.

Previous studies from our lab demonstrated DVD-deficiency induced alterations in pup vocalisations [11]. Pup vocalisations are thought to influence maternal care, a factor we chose to investigate in more detail here. Similarly, we have previously shown DVD-deficiency induces some subtle alterations in social play behaviour in juveniles [11]. Here we chose to investigate social behaviour, but we also examined the degree of self-directed stereotyped behaviour when in the presence of a conspecific.

In addition, a growing number of studies in patients and animal models of autism have demonstrated an association between autism and gastrointestinal imbalances such as altered gut microbiome composition [14–18], faecal short chain fatty acid levels [19], increased gut permeability [20, 21], changes in villi architecture [22], and immune dysregulation [23–25]. Vitamin D-deficiency/vitamin D supplementation is also known to regulate the composition, and diversity of gut microbiome, levels of microbial metabolites, innate and adaptive immune responses in the gut, integrity of the gut epithelium and several aspects of gut health in experimental animals and humans [26–32]. Thus, our objective in this study was to examine if the gut microbiome and gut physiology were also altered by DVD-deficiency and whether any alterations were associated with ASD-related behavioural phenotypes.

## Methods

### Animals and breeding

A detailed description of how our DVD-deficiency model is produced and confirmation of vitamin deficiency in Sprague-Dawley (SD) dams has been described elsewhere [11, 33]. Faecal pellets were collected from the DVD-deficient and Control pregnant dams at E15 and stored at −80 °C for microbiome sequencing. The day the pups are born is designated postnatal day 0 (P0) and pups were weaned at P21. In an alteration to the protocol mentioned in our previous publications, dams remained on their respective diets until weaning and weanlings remained on the same diet as dams until P35. The timeline for the animal breeding and experimental outline are presented in supplementary Fig. S1. All the animal procedures performed in this study were approved by The University of Queensland Animal Ethics Committee (QBI/555/16#10). Vitamin D-deficiency was confirmed by measuring the levels of 25-hydroxyvitamin D_3_ (25-OHD) in the serum collected from the pregnant dams (control = 25.8 ± 13.1 nM, deficient = 7.22 ± 12.0 nM) (*W* = 275, *p* = 0.00018, Wilcoxon rank test) and P35 offspring (control = 23.1 ± 10.7, deficient = 0.865 ± 1.23) (*W* = 929, *p* = 2.739e^−09^, Wilcoxon rank test).

### Maternal behaviour

Maternal behaviour was observed daily from P2 to P6 in the home cage. A camera was fitted to the top of the cage to record the activities of the whole litter (dams plus pups). Each litter underwent behavioural observations for two one-hour sessions per day. Thus, the total number of observations for each litter was: 5 postnatal days ×2 sessions per day = 10 observations. The number of maternal behaviours such as Licking/grooming and Arched-Back Nursing during the 10 sessions were recorded. Maternal behaviours were scored following the protocol of Franks et al. [34].

### Ultrasonic vocalizations (USVs) and pup retrieval task

Isolation-induced USVs were recorded from each pup at P7 and P9. On the test day, the pups were separated from their dam and placed in a surgical recovery chamber, maintaining a temperature of 34 ± 1 °C. The pups were subsequently placed in a sound-attenuated chamber one by one. The microphone was placed about 10 cm above the head of the pups and USV recordings were obtained using UltraVox XT system (Noldus Information Technology, The Netherlands). USVs were measured for three minutes, then pups were returned to the recovery chamber. Detector outputs were analyzed with UltraVox XT (3.0.80) software (see supplementary Table S1 for criteria used for valid USV calls).

The pup retrieval task was performed immediately after recording USVs for each litter. Initially, all the pups were placed on the opposite corner of the home cage (dimension: 57 cm × 39 cm × 20 cm) away from the nest. Then, the dam was introduced into the centre of the cage and video recorded. Recordings were stopped when the dam retrieved all the pups to the nest or after 10 min had elapsed. Latency to retrieve the first, second, third, fourth and fifth pup was recorded.

### Adolescent social play behaviour

Adolescent offspring between P35 to P40 were tested for social play (rough and tumble) behaviour. The apparatus consists of a novel chamber (length 52 cm, breadth 36 cm) containing 2 cm deep wood chip bedding. Rats were habituated (day 1) by placing body weight and sex-matched littermates in pairs in the testing chamber for 30 min. On the test day (day 2), the same littermate pairs were placed together back in the testing chamber and video recorded for 10 min. During the test, the animals are allowed to interact freely. Parameters of social play behaviours such as latency to interact, frequency of pouncing, pinning and total play duration were recorded. The data were analysed using Observer software (Noldus Information Technology, The Netherlands) [35]. Littermate pairs were considered as a single experimental data point for the behavioural analysis.

### Repetitive self-grooming behaviour

To assess repetitive behaviour in the adolescent rats, the same recordings used for social play behaviour were further scored for self-grooming behaviour using Noldus Observer software. Number and duration of bouts of self-grooming in which an animal groomed any part of its own body were scored [36]. Allo-grooming, in which an animal grooms any body part of the conspecific was also scored.

### Tissue collection and evaluation of gut microbiome and gut physiology

After social play behaviour was completed, 32 adolescent animals (8 control males and 8 DVD-deficient males and 8 control females and 8 DVD-deficient females) were injected with poly(I:C) (a synthetic double-stranded viral RNA) (dose: 5 mg/kg body weight). A replicate cohort of the same groups was injected with saline vehicle. Four hours after injection, animals were euthanised and gut tissues collected. Poly(I:C) was used to induce inflammation to establish whether if inflammatory response was altered by DVD-deficiency. The samples collected include blood, jejunum, ileum, and colon tissues. From the saline-exposed animals, gut contents were collected from colon for gut microbiome composition and ileum for short chain fatty acids. The proximal colon tissues were used for quantitative real-time polymerase chain reaction (qPCR).

### Gut microbiome

Microbial DNA was extracted from the colon contents of P35 animals (*N* = 32) using DNeasy^®^ Powersoil^®^ Pro kit (QIAGEN) and sent to the Australian Centre for Ecogenomics (ACE) for 16S rRNA gene amplicon sequencing. The sequencing was performed by amplification of the V3-V4 region of 16S rRNA using a barcoded primer set 16S 341F/806R (Forward:5′-CCTACGGGNGGCWGCAG-3′; Reverse: 5′-GACTACHVGGGTATCTAATCC-3′). Paired-end, 2 × 300 bp sequencing was performed on an Illumina platform at ACE. The raw demultiplexed fastq files obtained from the sequencing centre were analysed by Quantitative Insights into Microbial Ecology II (QIIME2) version 2021.2 software pipelines [37]. The sequences were matched to SILVA reference database, release 138.1(https://www.arb-silva.de/download/arb-files/) for determining bacterial taxonomy. For the statistical analysis of the gut microbiome data, MicrobiomeAnalyst ([38] and Phyloseq R package were used. To compare the microbiome differences between DVD-deficient and Control animals, alpha and beta diversity were determined. Alpha diversity represents the quantity of bacterial species present in a sample whereas beta diversity measures the differences in the gut microbiome between different samples [39]. Alpha diversity measures were determined by Observed species (bacterial Richness), Simpson index (Evenness), Shannon index and Chao1 indices. Richness refers to the presence or absence of organisms in a given environment whereas Evenness takes into account the abundance of the organisms. The Shannon index considers both number of organisms and their relative abundance. Chao1 is a qualitative measure of alpha diversity like richness but with an emphasis on rare species. Beta diversity was measured by Principal Co-ordinate Analysis (PCoA) plots and two measures were used: Bray-Curtis (non-phylogenetic) and Weighted Unifrac (phylogenetic). In addition, faecal samples from the pregnant dams were also collected and analysed similarly.

### Measurement of short-chain fatty acids

Ileal contents were recovered, and short-chain fatty acids (SCFAs) extracted in 50% acetonitrile, vortexed, centrifuged and supernatant analysed using LC-MS at Metabolomics Australia, UQ, Brisbane. Ileal samples were selected from the same animals that were used for microbiome sequencing.

### Gut histology

Ten cm of jejunum was collected and fixed in 10% buffered formalin. The jejunal tissues were processed using the Swiss Roll Technique [40]. Briefly, a 5-µm section was cut using a microtome and stained with Haematoxylin and Eosin. A single section from each animal was examined using light-microscopy for villi length, lymphocyte and goblet cell number by applying the protocol developed by Erben et al. [41]. Stereo-investigator^®^ software (MBF Bioscience, US) was used for unbiased counting site selection. Gut tissues from males only were used for the histological examination.

### Inflammatory cytokines

A separate 2 cm piece of ileum was homogenised in MSD lysis buffer (1:3 w/v) containing a protease inhibitor (complete mini EDTA-free, Roche) and was used for both total protein and IL-6 and TNF-α assay (Pro-inflammatory rat panel 1, Meso Scale Discovery, Rockville MD USA). Maternal sera were similarly analysed (but unlike ileum, sera were used directly without homogenization).

### Quantitative real-time PCR (qPCR)

Total RNA was extracted from the proximal colon tissues by homogenizing in Trizol reagent (Invitrogen) using a Polytron(IKA®ULTRATURRAX^®^). cDNA synthesis was performed by using SensiFAST^TM^ cDNA Synthesis Kit (Bioline, UK) according to the manufacturer’s protocol. qPCR was performed by using SensiFAST^TM^ SYBR No-ROX kit master mix (Bioline, UK) in Roche LightCycler480 Thermalcycler (Roche Life Science). All expression levels were normalized to that of endogenous control glyceraldehyde-3-phosphate-dehydrogenase (gapdh) and results were analysed using the comparative threshold method. For primer and RT-PCR protocol details, see supplementary Table S2.

### Statistical analysis

Behaviours were analysed by multivariate analysis of variance (MANOVA) using SPSS version 27, Chicago, IL, USA to examine the effects of maternal diet, sex and maternal diet × sex interactions. For pup retrieval, a mixed effect model was used to analyse the effect of DVD-deficiency on repeated measures for sequential pup retrieval. Prior to statistical analysis, potential outliers were checked using Grubb’s test [42] and any observation with a Zscore of 4.0 and above were considered as outlier and removed. Data are represented as 25th percentile, median and 75th percentile values and statistical significance was established at an alpha of 0.05. For gut tissue analysis, MANOVA was used to examine the effect of maternal diet, sex, Poly(I:C), and diet × sex × Poly(I:C) interactions. For correlation between bacterial abundance and pouncing behaviour, the top six bacteria that showed nominally significant correlations in either DVD-deficient or Control groups are shown. For all behavioural, biochemical and microbiome analyses, animals were recoded to blind experimenter to group. Cage effect was checked using two-way ANOVA taking cage as independent variable and the frequency of pouncing (the most prominent behavioural finding in P35 animals in our study) as dependent variable. The analysis showed that there was no significant cage effect on the behavioural outcomes (Table S5).

## Results

### DVD-deficiency alters maternal and pup behaviours

**Fig. 1:**
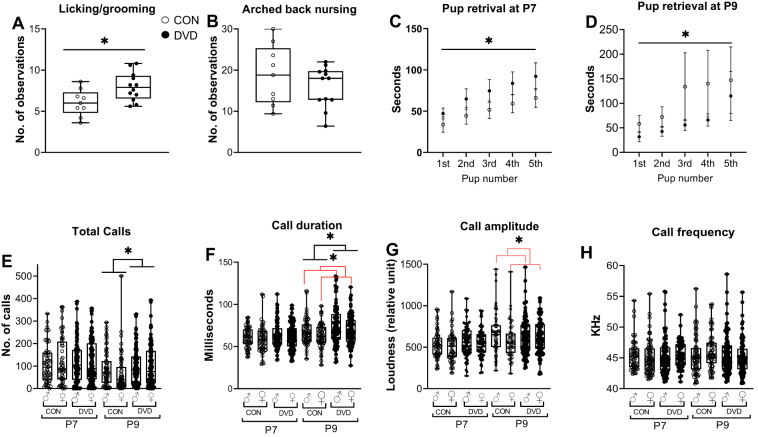
DVD-deficiency alters both maternal and pup behaviour. Maternal behaviour was video-recorded and observed from P2 to P6. **A** DVD-deficient dams showed increased licking/grooming (LG) compared to control dams (*F*_1,21=2.625_, *p* < 0.05). **B** There was no effect of DVD-deficiency on arched-back nursing. The circles represent individual dams. **C**, **D** Maternal retrieval of the pups was tested at two time points (P7 and P9) immediately following USV measurement. Data reported as latencies to retrieve the first, second, third, fourth and fifth pup (in seconds). Analysis was done using a mixed effects model. Overall DVD-deficient dams retrieved their pups slower than the control dams at P7 (*F*_1,90=6.950_, *p* = 0.0099), whereas this was reversed at P9 (*F*_1,85=3.963_, *p* = 0.049). DVD dams *n* = 12, CON dams *n* = 8 at P7. DVD dams *n* = 11, CON dams *n* = 8 at P9. *p < 0.05, **p < 0.01. Error bars show SEM. **E**–**H** Measurement of pup’s USVs at two time points (P7 and P9) in a brief isolation from their dams. No significant differences were found in the four USV parameters at P7. However, DVD-deficient pups exhibited greater number (**E**) and longer duration (**F**) of calls at P9 compared to control pups. There were no differences in the call amplitude (**G**) and call frequency (**H**) at P9 between DVD-deficient and control pups. At P9, male pups emitted longer duration of calls (**F**) and louder calls (**G**) compared to female pups. DVD males *n* = 89, DVD females *n* = 93, CON male *n* = 64, CON females *n* = 59. *p < 0.05. Black lines differences by diet, red lines difference by sex. CON Control, DVD DVD-deficient. The lower boundary of the box plot indicates 25th percentile, the middle line median and upper boundary indicates 75th percentile.

DVD-deficient dams showed increased Licking/grooming *F*_1, 21_ = 2.625, *p* < 0.05) compared to control dams. However, there was no effect of DVD-deficiency on Arched back nursing (*F*_1,21_ = 1.023, *p* > 0.05) (Fig. 1A, B). Pup retrieval was altered by DVD-deficiency (Fig. 1C, D). At P7, DVD-deficient dams took longer to retrieve pups (*F*_1,90=6.950_, *p* = 0.0099), compared to control dams. However, this was reversed at P9 (*F*_1,85=3.963_, *p* = 0.049).

At P7 no pup USV parameter was altered by DVD-deficiency or sex. However, at P9, DVD-deficient pups exhibited a significantly greater number (*F*_1, 305_ = 6.194, *p* < 0.05) and a longer duration of calls (*F*_1, 305_ = 5.692, *p* < 0.05) compared to control pups. Also, male pups produced a longer duration of calls (*F*_1, 305_ = 6.753, *p* < 0.05) and higher amplitude calls (*F*_1, 305_ = 4.669, *p* < 0.05) compared to female pups at P9 (Fig. 1E–H). There was no effect of sex on total calls (*F*_1,305_ = 0.988, *p* = 0.321) and call frequency (*F*_1,305_ = 0.687, *p* = 408) at P9.

### DVD-deficiency decreases adolescent social play behaviours and increases self-grooming

**Fig. 2:**
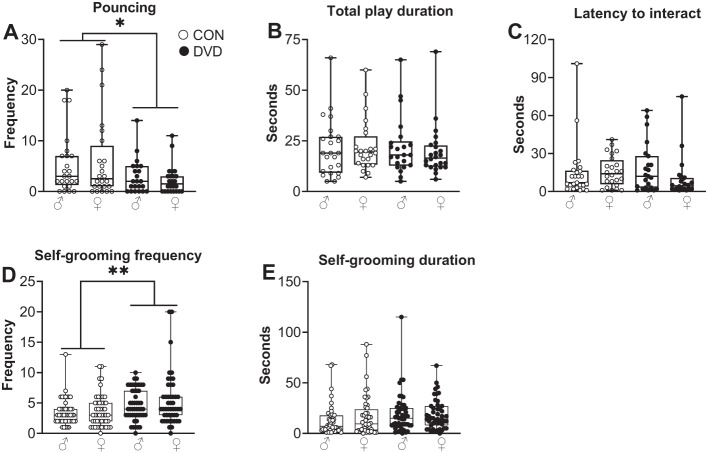
DVD-deficiency decreases social play and increases stereotyped behaviour in adolescent rats. **A**–**C** Social play behaviour was measured in P35 offspring. (A)DVD-deficient offspring showed significantly less pouncing compared to control offspring (*F*_1, 94_ = 7.328, *p* = 0.008). No differences were found in (**B**) total play duration or (**C**) latency to interact. There was no main effect of sex on frequency of pouncing (*F*_1,94_ = 0.006, *p* = 0.939), total play duration (*F*_1,94_ = 0.074, *p* = 0.787) and latency to interact (*F*_1,94_ = 1.123, *p* = 0.292). CON males *n* = 24, CON females *n* = 24, DVD males *n* = 24, DVD females *n* = 24. **D**, **E** Self-grooming behaviour was analysed from social play recordings. **D** DVD-deficient rats showed increased bouts of self-grooming compared to controls (*F*_1,191_ = 11.595, *p* = 0.001). **E** There was no difference in the duration of self-grooming between DVD-deficient and control animals (*F*_1,191_ = 2.823, *p* = 0.095). CON males = 48, CON females = 48, DVD males = 48, DVD females = 47. **p < 0.01.

DVD-deficient adolescent rats showed reduced frequency of pouncing compared to control rats (*F*_1, 94_ = 7.328, *p* = 0.008). However, there was no effect of DVD-deficiency on total play duration (*F*_1,94_ = 0.108, *p* = 0.743) or latency to interact (*F*_1,94_ = 0.118, *p* = 0.732). There was no main effect of sex or diet x sex interaction on frequency of pouncing (*F*_1,94_ = 0.006, *p* = 0.939), total play duration (*F*_1,94_ = 0.074, *p* = 0.787) and latency to interact (*F*_1,94_ = 1.123, *p* = 0.292) (Fig. 2A–C). The number of “pinning” events was quite low therefore the presence or absence of pinning rather than the number of pinning events was analysed by chi-square test. We show that DVD-deficiency was associated with reduced frequency of pinning (χ^2^_1,94_ = 5.361, *p* = 0.021) (Table 1). There was no association of sex with pinning (χ^2^_1,94_ = 0.812, *p* = 0.368).

**Table 1:** Chi-square test of independence for pinning behaviour in adolescent offspring.

Diet	Animals showing pinning *n*	Animals not showing pinning *n*	Sample size *N*	Chi-square test of independence
CON	13	35	48	χ^2^_1,94_ = 5.361, *p* = 0.021
DVD	4	42	46	

DVD-deficiency was associated with a lower occurrence of pinning (χ^2^_1,94_ = 5.361, *p* = 0.021). There was no association of sex with pinning (χ^2^_1,94_ = 0.812, *p* = 0.368). CON = Control, DVD = DVD-deficient. Control males *n* = 24, Control females *n* = 24, DVD males *n* = 24, DVD females *n* = 24, *p < 0.05.

In the presence of a conspecific, DVD-deficient rats showed increased frequency of self-grooming compared to control rats (*F*_1,191_ = 11.595, *p* = 0.001). However, there were no differences in the duration of self-grooming between DVD-deficient and control animals (*F*_1,191_ = 2.823, *p* = 0.095) (Fig. 2D, E). Sex had no effect on both frequency of self-grooming (*F*_1,191_ = 1.128, *p* = 0.290) and duration of self-grooming (*F*_1,191_ = 0.464, *p* = 0.497). There was also no diet × sex interaction. Allogrooming events were quite low, therefore the presence or absence of allogrooming rather than the number of allogrooming events was analysed by chi-square test. We show that neither diet (χ^2^_1,191_ = 0.631, *p* = 0.427) nor sex (χ^2^_1,191_ = 0.007, *p* = 0.933) had any association with allogrooming (Table 2).

**Table 2:** Chi-square test of independence for allogrooming behaviour in DVD-deficient offspring.

Diet	Animals showing allogrooming (Yes)	Animals not showing allogrooming (No)	Sample size *N*	Chi square test of independence
CON	38	58	96	χ^2^_1,191_ = 0.631, p = 0.427
DVD	43	52	95	

DVD-deficiency was not associated with allogrooming. Sex was also not associated with allogrooming (χ^2^_1,191_ = 0.007, *p* = 0.933). Control males *n* = 48, Control females *n* = 48, DVD males *n* = 48, DVD females *n* = 47.

### DVD-deficiency alters gut microbiome and this correlates with social behavioural changes in offspring

**Fig. 3:**
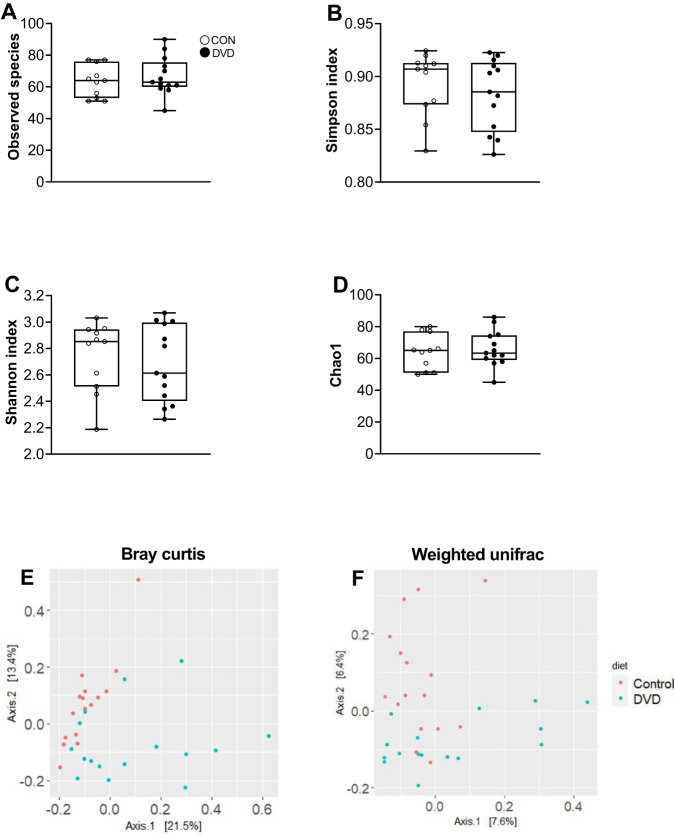
Alpha and beta diversity measures in the DVD-deficient and control offspring. All results are presented at genus level. **A**–**D** Alpha diversity. Observed species, Simpson’s index, Shannon index and Chao1 were not significantly different between DVD-deficient and control animals. **E**, **F** Measures of Beta diversity between DVD-deficient and control offspring. Differences in the microbial composition between DVD-deficient and control animals were determined by Bray Curtis (**E**) and Weighted Unifrac (**F**). Bray Curtis (ANOSIM, *R* = 0.196, *P* = 0.001), Weighted Unifrac (ADONIS, *R*^2^ = 0.048, *P* = 0.001). CON *n* = 16, DVD *n* = 16.

Colon contents from P35 offspring were used for the gut microbiome analysis. An average of 21,101 sequences were obtained from each of the 32 samples. The results are presented at genus level for alpha and beta diversity. There were no differences in the alpha diversity measures between DVD-deficient and control offspring (Fig. 3A–D. In contrast, analysis of beta diversity showed significant clustering of the animals into DVD-deficient and control diets (Bray Curtis distance: *R* = 0.196, *p* = 0.001) (Fig. 3E). Beta diversity based on the measurement of phylogenetic distances between the taxa also clearly showed distinct microbiome composition by diet (Weighted Unifrac: *R*^2^ = 0.048, *p* = 0.001) (Fig. 3F). DVD-deficiency did not result in alteration of alpha and beta diversity in the pregnant dams (Fig. S2).

In respect to individual species, we found four bacteria (*Akkermansia, Fusicatenibacter, Allobaculum and Turicibacter*) whose relative abundance in the offspring was significantly altered by DVD-deficiency (see supplementary Fig. S3).

**Fig. 4:**
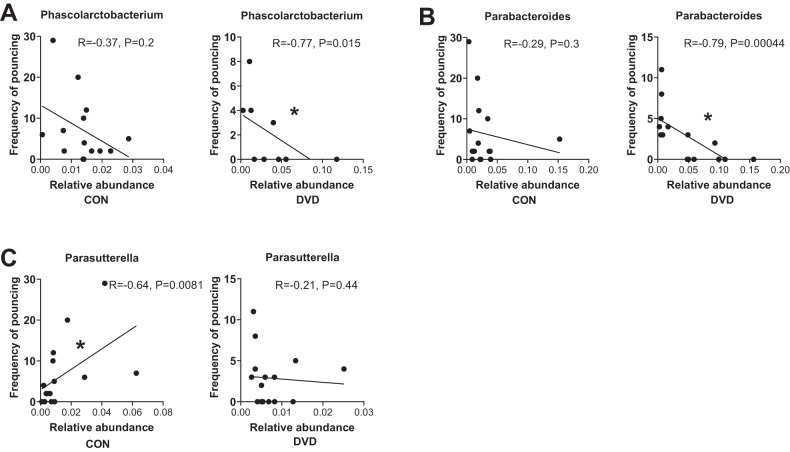
Spearman correlations between gut bacterial genera with social play behaviour (frequency of pouncing). The top correlations for any genus with pouncing behaviour in either DVD-deficient or control offspring are depicted. There was a negative correlations between *Phascolarctobacterium* (*R* = −0.77, *p* = 0.015) (**A**) and *Paracteroides* (*R* = −0.79, *p* = 0.00044) (**B**) with the frequency of pouncing in DVD-deficient group, but not in control. The genus *Parasutterella* was also negatively correlated with frequency of pouncing in controls but not in DVD-deficient group (*R* = −0.64, *p* = 0.0081) (**C**). CON *n* = 16, DVD *n* = 16, *p < 0.05. The small circles represent individual samples. The *p*-values shown for each bacterium genus are nominally significant values, not adjusted for multiple correction.

In respect to behaviour, we found a negative correlations between *Phascolarctobacterium* (*R* = −0.77, *p* = 0.015) (Fig. 4A) and *Paracteroides* (*R* = −0.79, *p* = 0.00044) (Fig. 4B) with the frequency of pouncing in DVD-deficient group, but not in control. The genus *Parasutterella* was also negatively correlated with frequency of pouncing in controls but not in DVD-deficient group (*R* = −0.64, *p* = 0.0081) (Fig. 4C).

### DVD-deficiency increases ileum propionate levels

**Fig. 5:**
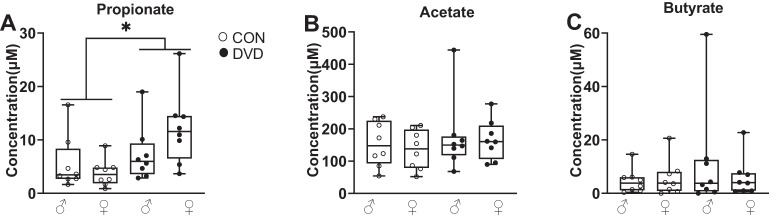
Measurement of three SCFAs in the ileum of the offspring. DVD-deficiency increased propionate (**A**) levels but did not affect the levels of acetate (**B**) or butyrate (**C**). CON male *n* = 8, CON female *n* = 8, DVD male *n* = 8, DVD female *n* = 8. *p < 0.05.

There was an increased propionate level in the ileum of DVD-deficient animals compared to controls (*F*_1,32_ = 7.609, *p* < 0.05). However, there were no differences in the acetate (*F*_1,32_ = 0.873, *P* = 0.358) and butyrate (*F*_1,32_ = 0.821, *p* = 0.373) levels. There was no effect of sex on the levels of acetate (*F*_1,32_ = 0.247, *p* = 0.623), propionate (*F*_1,32_ = 0.680, *p* = 0.416) and butyrate (*F*_1,32_ = 0.319, *p* = 0.577) (Fig. 5).

### DVD-deficiency effects on gut physiology

**Fig. 6:**
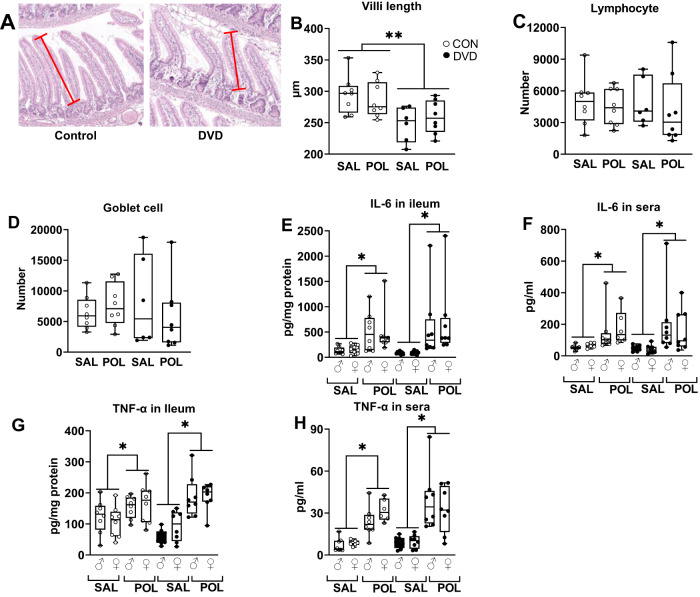
DVD-deficiency decreases gut villi length but has no effect on gut inflammatory cytokines. **A**–**D** Histological examination of jejunal sections of P35 offspring. Jejunal tissues were collected four hours after the animals were injected with either saline or poly(I:C). **A** A 5 µm thick section was prepared by Swiss roll technique. **B** DVD-deficiency resulted in decreased villi length, but there was no effect of poly(I:C) or diet × poly(I:C) interaction on villi length. DVD-deficiency had no effect on lymphocyte (**C**) or goblet cell number (**D**). SAL = Saline, POL = Poly(I:C). CON SAL *n* = 8, CON POL *n* = 8, DVD SAL *n* = 6, DVD POL *n* = 8. Only males were used for the gut histology analysis. **p < 0.01. **E**–**H** Inflammatory cytokines were measured in the P35 offspring ileum and sera. As expected, compared to a vehicle injection, Poly(I:C) clearly elevated the levels of both IL-6 and TNF-α in ileum and sera (*p* < 0.05). There was no main effect of DVD-deficiency on the levels of IL-6 (**E**, **F**) and TNF-α (**G**, **H**) in ileum and sera. However, there was interaction of diet × Poly(I:C) on TNF-α levels in ileum (*F*_1,64_ = 7.394, *p* = 0.009). SAL = Saline, POL = Poly(I:C). CON SAL *n* = 16, CON Poly(I:C) *n* = 16, DVD SAL *n* = 16, DVD PoL *n* = 16. *p < 0.05.

DVD-deficient animals had decreased villi length compared with control animals (*F*_1,30_ = 13.072, *p* = 0.001) (Fig. 6B). There was no effect of acute poly (I:C) on villi length (*F*_1,30_ = 0.018, *p* = 0.896) (Fig. 6B). In addition, we did not find a main effect of DVD-deficiency on lymphocyte count (*F*_1.30_ = 0.049, *p* = 0.827)(Fig. 6C) or goblet cell count (*F*_1,30_ = 0.003, *p* = 0.958) (Fig. 6D) nor any effect of Poly(I:C) on goblet cell (*F*_1,30_ = 0.097, *p* = 0.758) or lymphocyte number (*F*_1,30_ = 0.473, *p* = 0.498). Moreover, we found reduced mRNA expression of ZO-1 in the colon from DVD-deficient offspring compared to control offspring (*F*_1,63_ = 4.523, *p* = 0.038) (see Supplementary Fig. S4).

As expected, Poly(I:C) signifcantly elevated the levels of both IL-6 and TNF-α in ileum and sera, compared to saline treatment (*p* < 0.05) (Fig. 6E–H). There was no main effect of sex on sera IL-6 (*F*_1,64_ = 0.000, *p* = 0.984) and TNF-α (*F*_1,64_ = 0.132, *p* = 0.718); or ileum IL-6 (*F*_1,64_ = 0.056, *p* = 0.814) and TNF-α (*F*_1,64_ = 0.394, *p* = 0.533). There was also no effect of DVD-deficiency on the levels of IL-6 and TNF-α in ileum and sera. However, a significant interaction of diet × Poly(I:C) on TNF-α levels in ileum (*F*_1,64_ = 7.394, *p* = 0.009) was found, showing greater response to Poly (I:C) in DVD-deficient animals (Fig. 6G).

## Discussion

The DVD-deficiency animal model produces deficits in pup-dam communication, increased stereotyped behaviours and impaired social interaction, all behavioural phenotypes of relevance to autism. Correlations between gut microbiome and certain social behaviours raise the potentially interesting question that this developmental risk factor may increase the risk for autism via the gut microbiota or alterations to gut health.

### DVD-deficiency alters maternal and offspring behaviour relevant to autism

An earlier study from our lab, showed increased corticosterone release in response to stress in DVD-deficient dams [43]. Maternal stress is an established risk-modifier for autism [44, 45]. Proper maternal care towards their offspring is essential for the development of normal social behaviour in animals and humans [46]. The increased licking/grooming observed in DVD-deficient dams in our study may reflect a heightened state of anxiety or stress in DVD-deficient dams and pups as maternal stress is correlated with the frequency of such grooming in rodents [47]. Variation in maternal care in rodents has been shown to affect the development of neural systems that mediate stress reactivity, which in turn could influence maternal behaviour. For example, offspring born to high licking/grooming dams show decreased cortico-releasing factor mRNA expression in the central nucleus of the amygdala [48]. The enhanced pup retrieval, at least at the later time point, may also be consistent with enhanced maternal anxiety.

To examine how pups respond and communicate to rat dams, we further measured USVs in the pups. A rudimentary early communication in pups is the USVs they emit when separated from their dam [49]. DVD-deficiency influenced USVs at P9, with DVD-deficient pups emitting increased numbers and longer duration of calls at P9. These findings are consistent with two recent studies showing increased USVs in DVD-deficient rats at P9 [11] and P12 [50]. Many other animal models of autism have also demonstrated altered USVs (either increased or decreased) [51–53]. However, the mechanisms involved in USVs remain poorly defined. One hypothesis implicates dopamine (DA) systems. The DA D2 receptor knock-out mouse shows reduced isolation-induced USVs [54] and consistent with this, elevating DA levels using amphetamine increased USVs in adult rats [55]. Our laboratory has consistently shown that DVD-deficiency delays DA neuron development in the embryonic brain [9, 10, 56–59]. Whether DA development is linked with pup’s USVs remains a topic of ongoing interest.

In terms of understanding maternal behaviour at the ages where USVs were recorded, DVD-deficiency enhanced both pup USV number and call duration at P9. This corresponded with faster pup retrieval by dams. Pup USVs are important trigger for pup retrieval by their dams [60]. Increased USVs may represent a heightened state of anxiety or stress in DVD-deficient pups. Studies have shown that the administration of anxiety-inducing drugs results in increased USVs in mouse pups [61]. This suggest that at this age at least, pup USVs may drive maternal behaviour. At P7 however DVD-deficient dams were slower to retrieve pups yet there was no alteration in pup USVs suggesting some other mechanism.

Deficits in social skills and abilities are diagnostic features of autism [62]. In rodents, this is measured by adolescent social play [63] and adult social interaction assays [64]. Social play behaviour such as “pouncing and pinning” is a vigorous form of social interaction commonly observed in young mammals [36]. This behaviour is highly prevalent in adolescent rats [35]. It is highly rewarding and believed to play an essential role in social and cognitive development. Manipulation of DA systems results in robust changes in social play behaviour in rodents [65]. Given a large number of animal studies now demonstrate vitamin D levels in the developing brain affect the ontogeny of DA neurons, DA release in adults and DA-mediated behaviours [9, 10, 43, 56, 58], this could be one mechanism at play for the reduced social play seen in DVD-deficient P35 animals.

We also elected to examine the amount of self-grooming an animal would engage in whilst in the presence of a conspecific during social play. Self-grooming is a rodent behaviour considered to have some face validity to the repetitive behaviours observed in autism [66]. This is generally assessed by examining an animal in isolation from cage mates [36, 67, 68]. However, we argue that our measure during social interaction may more closely reflect a stereotyped or “inward directed” activity when a social stimulus was present. The P35 DVD-deficient animals in our study showed higher frequency of self-grooming when in the presence of a conspecific, consistent with their diminished social interaction. Several genetic and environmental models of autism in rodents demonstrate increased self-grooming behaviours. The brain specific mechanisms involved in regulation and maintenance of self-grooming remains unclear [69]. Pharmacological studies have suggested a balance between dopamine D1 and D2 receptor systems in the regulation of self-grooming [70]. Some other studies have also reported involvement of glutamate, as anti-glutamatergic agents have been shown to induce grooming in rodents [71]. Given our previous study showing alterations of dopamine and glutamine concentrations in DVD-deficient neonatal rat brain [59], further investigation to examine the involvement of these neurotransmitter systems in self-grooming behaviour is needed.

### DVD-deficiency alters gut health-associated outcomes relevant to autism

Faecal samples were collected from pregnant DVD-deficient dams and their adolescent offspring to examine if DVD-deficiency alters gut microbiome. No differences in gut microbiome (alpha and beta diversity) were found between DVD-deficient and control dams. In P35 offspring, although alpha diversity within each experimental group was unchanged at the genus level, beta diversity determined by principal coordinate analysis clearly showed differences between dietary groups indicating that the composition of the microbiome is altered by the lack of vitamin D.

When we examined the top correlates between bacterial species and the major behaviour examined at P35 (pouncing), bacterial abundance correlated reversibly for three bacteria: *Phascolactobacterium, Parabacteroides* and *Parasutterella*. There are mixed findings in literature on whether these bacteria are increased or decreased in autism/animal models of autism. The abundance of *Clostridium* was increased in autistic children in one study [72]. Studies suggest *Clostridium species* likely regulate development and function of regulatory T cells in the intestine [73], however, we did not find this bacteria correlated to pouncing behaviour in the current study. Strati et al. found that *Parabacteroides* was decreased in autistic children [74], whereas a study by Finegold et al. [75] showed *Parabacteroides* was increased in autism. Variations in diet or behaviour are highly likely to affect regional gut microbiome outcomes. Until such issues can be appropriately controlled, the emerging links between gut health and psychiatry will continue to produce conflicting outcomes [76, 77].

It is generally accepted that a large proportion (about 50%) of infant gut microbiome is derived from the mother’s gut through vertical transmission, although there are studies showing contribution of microbiome from other maternal sites such as skin, vagina and oral cavity [78, 79]. Our findings show that although DVD-deficiency didn’t alter the maternal gut microbiome composition, there was differences in the gut microbiome between DVD-deficient and control offspring. A number of factors may explain this observation and elaborate its implication. Firstly, the neonatal gut is microbially naïve and the gut mucosa (and associated immune system) are still developing [80]. Given vitamin D is an important immune regulator [81] perhaps the gut is immunologically compromised in the DVD-deficient pups perhaps allowing a different level of host resistance to initial microbial colonization. Secondly, variations in the quality of maternal care after birth have been shown to influence the composition of the infant gut microbiome in rat offspring [82]. This may indicate that the increased maternal licking/grooming, altered rates of pup retrieval and increased pup call number and length which are all altered by DVD-deficiency might affect initial microbial seeding in the pup’s gut.

Studies in preclinical models however are more consistent. New studies continue to reveal associations between gut microbiome and social behaviour [83, 84]. Gut biomes from patients with autism when transferred to animals produce deficits in social behaviours [16]. In respect to individual bacteria, Hsiao et al. showed that administration of *Bacteroides fragilis*, an intestinal commensal bacteria, into maternal-immune activated mice restored social behaviours and USV deficits in adult offspring [20]. The bacterium *Enterococcus faecalis* has been shown to affect host social behaviour by regulating certain stress responsive neurons in the brain [85]. Thus, gut microbiome could be an important regulator of the gut microbiota-brain axis affecting host behaviour and brain function. We did show an increase in the relative abundance of *Akkermansia* and *Turicibacter* in DVD-deficient offspring whereas decreased abundance of *Fusicatenibacter and Allobaculum* in DVD-deficient offspring compared to control offspring (see supplementary Fig. S3). *Akkermansia* has been previously reported to be elevated in autism and an important organism having a role in maintaining gut mucous membrane integrity [72]. *Allobaculum* is a SCFA producer and one study links its role in lipid metabolism and cardiovascular disease [86]. Thus, vitamin D may also regulate gut function by influencing the population of these bacteria.

Alterations in faecal levels of SCFAs have been reported in autistic children [87, 88]. SCFAs (mainly acetate, propionate and butyrate) are microbial metabolites released by the fermentation of the non-digestible nutrient polymers in the gut. SCFAs are thought to regulate metabolism, immune function, gut integrity and are also believed to be central to any proposed CNS effects from altered gut physiology [89, 90]. The increased propionate levels in ileum found in DVD-deficient offspring may reflect enhanced activity of SCFA producing phyla such as Firmicutes. However, the relative abundance of the Firmicutes was not different between DVD-deficient and control animals in our study, suggesting other mechanisms operating in the gut. Interestingly, the genus *Akkermansia* is a SCFA producer [91]. Whether an increase in an *Akkermansia* species in DVD-deficient offspring is responsible for the increase in propionate levels is unknown. In rodents, supplementation of propionate to rats has been shown to induce repetitive self-grooming behaviours [92]. Thus, we checked to see if social interaction or self-grooming was associated with SCFAs; however, none of the three SCFAs correlated with either behaviour (see supplementary Tables S3 and S4). Usually, supplementation experiments involve relatively high dose of propionate (mg/kg range), thus it is not unexpected to see lack of behavioural correlates with very low propionate levels (in µM) as in our study.

Whether propionate would cause increased repetitive self-grooming behaviour or increased ultrasonic vocalization in the DVD-deficient offspring, supplementation of propionate (in the form of sodium propionate) to the control offspring is an appropriate experiment to further examine if this metabolite is causally linked to the repetitive behaviour/ultrasonic vocalization. However, propionate concentration measured in our animals are quite low (mean 7.23 µM) and we have not observed any correlation between the propionate levels and the self-grooming behaviour. Moreover, previous studies show that intraventricular infusion of relatively higher doses of sodium propionate (56 or 260 µM) is required to show significant behavioural outcomes in rats [93, 94]. Because of these reasons, the proposed supplementation experiment with propionate, may not produce any significant behavioural outcomes. We searched for the potential propionate producing bacteria in the literature and found that *Akkermansia municiphila* is identified as a propionate producer in the gut [95]. Interestingly, *Akkermansia* genus is significantly enriched in DVD-deficient group in our study, compared to control. This means that this bacterium probably is associated with increased propionate production in DVD-deficient animals and might also be associated with the increased self-grooming behaviour of the P35 offspring. However, due to the inherent limitation of 16 s amplicon sequencing, we could not resolve the taxa *Akkermansia* up to species level and hence could not confirm that the organism is in fact *Akkermansia municiphila*.

Our histological examination of the gut revealed that DVD-deficiency shortens the length of villi. There are very few studies that have specifically examined the effect of vitamin D on villi length. Birge and Alpers demonstrated that addition of the active vitamin D hormone to vitamin D-deficient rats resulted in 20% increase in villus length compared to deficient animals [96]. In a cell culture study using intestinal epithelial cells, vitamin D supplementation enhanced cell migration from crypts towards the apical villi [97]. This suggests that decreased villi length in DVD-deficient animals may reflect impaired cell migration from the crypts. Reduced villi length has also been reported in Shank3 Knockout mice (which is a genetic model of autism) [98]. DVD- deficiency was not associated with changes in the intraepithelial lymphocyte and goblet cell counts in our study. The effects of vitamin D are mediated by vitamin D receptor (VDR), which in turn, regulates the expression of several genes such as that of tight junction proteins (TJPs) [97, 99]. Zo-1 along with other TJPs regulate paracellular permeability of the gut [100]. One of the TJPs whose expression was significantly reduced in DVD-deficient offspring in our study is Zo-1. Although the finding was significant, this should be interpreted with caution as the effect seemed to have been driven primarily by Poly(IC) in DVD-deficient group.

Several studies have shown gut inflammation and reduced intestinal immune cells, in vitamin D-deficient or vitamin D-receptor knock out (VDR KO) animals. Vitamin D is an immune regulator, which suppresses inflammatory Th17 cells and induces the Treg cells, thus helping to maintain immunological homeostasis in the gut [32]. Studies examining the direct effect of vitamin D on goblet cells are scarce, but vitamin D-deficiency has been shown to cause thinner mucous layer in the gut and increase translocation of bacteria to the mesenteric lymph nodes predisposing the gut to inflammation. The thinner mucous layer could be related to the mucous degrading activity of genus *Akkermansia* [72]. Goblet cells secrete mucous, thus helping to protect the gut epithelium from bacteria and other toxic substances [101]. Although there was no change in the number of these immune relevant cells in the gut, further studies are required to examine the function of these cells and structure of the mucous layer in DVD-deficient offspring.

Our final experiment was the acute use of an immune activator Poly(I:C) to investigate whether the DVD-deficient gut was more vulnerable to pro-inflammatory agents. Vitamin D is a well-known immune regulatory agent [102] and we have shown that placental tissues from DVD-deficient dams have an enhanced inflammatory response to Poly(I:C) [12] As expected, DVD-deficiency did not induce any baseline inflammatory condition similar to what we report in placental tissue [12]. Also, as expected Poly(I:C) induced a robust increase in inflammatory markers in both P35 sera and gut tissue. However, unlike cultured placenta tissue in vitro, the acute (4 h) inflammatory response to Poly(I:C) in P35 offspring gut was unaffected by diet. Whether any long-term interactions between DVD-deficiency and exposure to Poly(I:C) at P35 remains unknown.

## Conclusions

Our primary goal in this experiment was to study whether gut microbiome or alterations to gut physiology are present in our animal model of DVD-deficiency. We again confirm that a range of ASD-related behaviours (altered pup-dam communication, reduced social interaction, and increased stereotyped behaviours) are present in this model and importantly social behaviour in DVD-deficient animals nominally correlates with the abundance of several bacterial species. Future studies could incorporate faecal microbiome transfers or colonisation with each of the implicated bacterial species in DVD-deficient offspring to establish causality. However, microbiota transfer experiments may be compromised by the altered behaviour of DVD-deficient dams as the increased licking and grooming and altered pup retrieval times which can adversely affect pup development independent of any effect of microbiome.

The epidemiological links between maternal vitamin D deficiency and increased autism risk [2, 5, 7, 103] are well-established. The emerging data concerning gut health in children with autism and the role of vitamin D in preserving gut function suggest this ASD-risk factor may act via alterations to the gut microbiota, gut SCFA synthesis or altering gut physiology. The high prevalence of vitamin D-deficiency in pregnant women [104–106] increases concern regarding this link.

## Supplementary information

List of bacteria altered by DVD-deficiency with FDR correction

Developmental vitamin D-deficiency produces autism-relevant behaviours and gut-health associated alterations in a rat model
