# Supplementary material for: Developmental vitamin D-deficiency produces autism-relevant behaviours and gut-health associated alterations in a rat model
Source: Transl Psychiatry. 2023 Jun 14;13:204. doi: 10.1038/s41398-023-02513-3 (PMC10267107; doi:10.1038/s41398-023-02513-3)
Supplement: Supplementary file 2 — Developmental vitamin D-deficiency produces autism-relevant behaviours and gut-health associated alterations in a rat model [file 41398_2023_2513_MOESM2_ESM.docx]

**Supplementary information**

**Developmental vitamin D-deficiency produces autism-relevant behaviours and gut-health associated alterations in a rat model**

**Figure S1. Experimental outline**

4 wk old female SD rat placed on diet

Mating with sires

Birth of pups

Social play behaviour, euthanized and tissue collection

Weaning

Maternal behaviour

P0

P2

P21

P9

P6

P7

P35

Week 10

USV and pup retrieval task


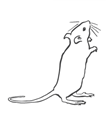


**Figure S2. Gut microbiome analysis between DVD-deficient and control pregnant dams.**

**Figure S3. Relative abundances of bacterial genera altered in DVD-deficient offspring compared to control offspring**

**Figure S4. Measurement of mRNA expression of Zo-1 in the offspring colon**

**Figure Legends**

**Figure S1.** Experimental outline of the animal breeding protocol and timeline for experiments. Four-week-old female Sprague Dawley (SD) rats are placed on either vitamin D-deficient or control diets for six weeks. At week 10, they are mated with sires on control diets and four pregnant dams housed together until gestational day 20 (E20), after which dams are housed singly for parturition. Observation of maternal behaviour begins at postnatal day 2 (P2), and behavioural testing completes at P35. After behavioural testing, some animals were injected (see text) with poly(I:C) or saline and after 4 hrs were euthanised and gut tissues, faecal matter and blood collected for the examination of gut microbiome, short-chain fatty acids, histology, qPCR and cytokine profiles.

**Figure S2.** Assessment of gut microbiome in the faecal samples in the pregnant dams. There was no difference in the alpha diversity (Fig.S2A-D) and beta diversity (Fig.S2E-F) between the DVD-deficient and control pregnant dams. Bray Curtis (ANOSIM, R=0.031, P=0.253), Weighted Unifrac (ADONIS, R^2^=0.058, P=0.226). CON n=11, DVD n=13.

**Figure S3**. The relative abundances of top four genera that are altered by DVD-deficiency are presented (after FDR correction). The abundances of *Akkermansia (A) and Turicibacter(B)* are increased in DVD-deficient offspring whereas those of *Allobaculum*(C) and *Fusicatenibacter* (D) are decreased in DVD-deficient offspring compared to control.

**Figure S4**. Expression of mRNA levels in proximal colon from the P35 offspring showing decreased relative expression of Zo-1 in DVD-deficient offspring compared to control offspring. The relative mRNA levels were normalized to housekeeping gene gapdh (glyceraldehyde -3-phosphate dehydrogenase). SAL= Saline, POL= Poly(I:C). CON SAL n=16, CON POL n=15, DVD SAL n=16, DVD POL n= 16.

**Table S1. Optimised criteria used for detecting valid USV calls in rat pups**

| Frequency | 40-60 kHz |
| --- | --- |
| Minimum amplitude | 110 |
| Minimum duration | 20 milliseconds |
| Gap between two calls | 10 milliseconds |

**Table S2. qPCR conditions and primer sequences**

qPCR was performed on a LightCycler 480 Thermal cycler (Roche Life Science) and the following PCR conditions were used: a denaturation step at 95 °C for 2 min and then amplification for 40 cycles (95 °C for 5 s, 62 °C for 10 s, and 72 °C for 20 s). Primer sequences are given below in the table.

| **Genes** | **Primer sequence** |
| --- | --- |
| Zona occluden1(ZO-1) | Forward-ATTCAGTTCGCTCCCATGAC  Reverse-GCTGTGGAGACTGTGTGGAA |
| Occludin (ocln) | Forward-CTACTCCTCCAACGGCAAAG  Reverse-AGTCATCCACGGACAAGGTC |
| Vitamin D receptor (vdr) | Forward-GGCTTCCACTTCAATGCTATG  Reverse-CATGCCGATGTCCACACAG |

**Table S3. Correlation of SCFAs with self-grooming behaviour in P35 offspring**

To check if the three SCFAs correlate with self-grooming behaviour, Pearson correlation was calculated. Results showed that SCFAs were not correlated with self-grooming behaviour of the offspring. Table S3A shows correlation statistics in control group and table S3B shows correlation statistics in DVD-deficient group.

**Table S3A**

|  |  | **Acetate** | **Propionate** | **Butyrate** |
| --- | --- | --- | --- | --- |
| **Self-grooming** | Pearson correlation | 0.429 | 0.407 | 0.348 |
|  | p-value | 0.098 | 0.117 | 0.187 |
|  | N | 16 | 16 | 16 |

**Table S3B**

|  |  | **Acetate** | **Propionate** | **Butyrate** |
| --- | --- | --- | --- | --- |
| **Self-grooming** | Pearson correlation | 0.353 | 0.304 | 0.339 |
|  | p-value | 0.180 | 0.252 | 0.199 |
|  | N | 16 | 16 | 16 |

**Table S4. Correlation of SCFAs with social play behaviour in P35 offspring**

To check if the three SCFAs correlate with social play behaviour (frequency of pouncing), Pearson correlation was calculated. Results showed that SCFAs were not correlated with social play behaviour of the offspring. Table S4A shows correlation statistics in control group and Table S4B shows correlation statistics in DVD-deficient group.

**Table S4A**

|  |  | **Acetate** | **Propionate** | **Butyrate** |
| --- | --- | --- | --- | --- |
| **Frequency of pouncing** | Pearson correlation | 0.254 | 0.049 | 0.090 |
|  | p-value | 0.342 | 0.857 | 0.740 |
|  | N | 16 | 16 | 16 |

**Table S4B**

|  |  | **Acetate** | **Propionate** | **Butyrate** |
| --- | --- | --- | --- | --- |
| **Frequency of pouncing** | Pearson correlation | -0.021 | -0.074 | 0.055 |
|  | p-value | 0.939 | 0.785 | 0.838 |
|  | N | 16 | 16 | 16 |

**Table S5**. Assessment of cage effect on the P35 behavioural outcomes. A two-way ANOVA test revealed that there was no significant cage effect on the frequency of pouncing in the P35 offspring.

|  | **Mean sq** | **F value** | **P-value** |
| --- | --- | --- | --- |
| Diet | 141.89 | 5.770 | 0.020* |
| Cage | 23.74 | 0.965 | 0.546 |

*Significant at alpha level of 0.05.
